# Effect of Temperature and Ceramization Atmosphere on the Structure and Microstructure of Boron-Modified SiBOC Materials

**DOI:** 10.3390/ma18081794

**Published:** 2025-04-14

**Authors:** Klaudia Łyszczarz, Piotr Jeleń, Patryk Szymczak, Maciej Sitarz

**Affiliations:** Faculty of Materials Science and Ceramics, AGH University of Krakow, al. Mickiewicza 30, 30-059 Kraków, Poland; lyszczar@agh.edu.pl (K.Ł.); patryk.szymczak@agh.edu.pl (P.S.); msitarz@agh.edu.pl (M.S.)

**Keywords:** SiBOC, pyrolysis, FT-IR spectroscopy, Raman spectroscopy, structure, microstructure, ceramization atmosphere

## Abstract

Boron-modified ceramic materials derived from polymers (PDC) are the subject of this research. The primary objective is to compare the structure and microstructure of SiBOC materials obtained in varying pyrolysis conditions in comparison to base SiOC materials. The preparation involved a number of stages, staring with the hydrolytic polycondensation method, followed by the initial thermal treatment and the final stage—pyrolysis process in argon or argon/hydrogen atmospheres at different temperatures. Bulk SiOC and SiBOC glasses were thoroughly analyzed. Microstructure studies included Scanning Electron Microscopy and Mercury Intrusion Porosimetry. Moreover, to confirm the structure, the research consisted of Fourier-Transform Infrared spectroscopy, Raman spectroscopy, and X-ray diffraction. The conducted research confirmed boron incorporation into the material structure in all samples. A free carbon phase has also been observed in SiBOC glasses, which has been confirmed in Raman spectroscopy measurements. This research indicates that in particular conditions, it is possible to obtain amorphous materials with nanocrystalline inclusions. This paper proves that the introduction of boron increases the porosity of materials and enhances their thermal stability.

## 1. Introduction

Silicon oxycarbide (SiOC)-based materials, also known as black glasses, have gained attention due to their unique properties and specific preparation method. Black glasses, which belong to the family of polymer-derived ceramics (PDC), exhibit a remarkable combination of mechanical strength and thermal and chemical stability, making them suitable for many applications. Their preparation consists of three main stages, and the final materials can take many different forms, such as bulk materials, coatings, foams, and filaments/fibers [1,2,3,4,5].

(1) Synthesis of preceramic precursors

The synthesis of these materials can be conducted using different methods. The most popular one is the sol–gel method, in other words hydrolytic polycondensation, which refers to two processes running in parallel—hydrolysis and polycondensation. This method allows for the easy modification of properties of final materials by appropriately selecting precursors that contain desired functional groups and/or cations [6,7,8]. Of particular interest are modifications with boron cations, in which the synthesis products are ladder-shaped silsesquioxanes doped with boron in the form of a sol [2,3,5,6,7,8,9].

(2) Initial thermal treatment in order to initiate the transformation from sol to gel.
(3) Pyrolysis—high-temperature treatment in an inert atmosphere, obtaining the final material.

Pyrolysis is a popular technique of sample preparation. In particular, its application in characterizing polymer materials deserves special attention. The process involves the rapid heating of the sample under controlled conditions of temperature and atmosphere [9].

Pyrolysis happens based on chemical reactions, which leads to the decomposition of organic compounds under high-temperature (400–800 °C [10]) and inert conditions [11]. From a practical point of view, it is not possible to conduct the pyrolysis process completely without oxygen, so the process occurs with a trace amount of oxygen [9,12].

During the pyrolysis process, organic compounds are transformed into gases or some liquids. Products in gaseous states include carbon, i.e., carbon dioxide, hydrogen, menthane, or other hydrocarbons [12].

It is worth mentioning that the pyrolysis process is similar to gasification, which uses partial oxidation to keep the thermal conditions the same; pyrolysis may also be carried out in anaerobic conditions using an external heat source [13]. This allows the process to be considered an effective way of processing and characterizing organosilicon materials [14,15,16].

Regarding the process of preparation, SiOC materials might be modified with different cations other than silicon. Boron-doped silicon oxycarbide (SiBOC) materials are worth noting due to their properties, such as high temperature and chemical resistance [1,2,17,18,19,20,21,22,23,24]. The properties of black glasses and their derivatives are related to their unique structure, including the free carbon phase [25]. This structural aspect causes silicon oxycarbide-based materials to exhibit a number of interesting properties, which (apart from the chemical composition) are mainly influenced by the method and conditions of their preparation. However, some characteristics are consistent across all silicon oxycarbide-based materials, which are thermal stability [26], high chemical resistance, and very good mechanical [27,28] and electrical properties [29,30,31,32,33,34,35]. Some other properties such as bioactivity and biocompatibility have also been described in another article [18].

The presence of Si-C bonds in silicon oxycarbide-based materials significantly enhances their chemical resistance. Compared to silica, they show greater resistance to hydrofluoric acid and alkalis. Additionally, the silicon–carbon bond prevents material from degradation.

Regarding the varying forms of the free carbon phase, which enhances electrical properties, silicon oxycarbide glasses can be used as either insulators or semiconductors. Their ability to conduct electricity is linked to the formation of a graphene network, with materials processed at higher temperatures displaying better conductivity than those treated at lower temperatures [31].

This article focuses on the structural and microstructural characterization of SiBOC materials in comparison to SiOC obtained by pyrolysis processes in higher than usual temperatures—like 1000, 1200, and 1400 °C—and in two different atmospheres—argon and argon/hydrogen [30,36]. Moreover, to investigate the exact transitions, e.g., carbothermal reduction and processes taking place in increasing temperatures as well as changing atmospheres.

## 2. Materials and Methods

### 2.1. Synthesis of SiBOC Materials’ Precursors

The synthesis of preceramic precursors has been conducted using the hydrolytic polycondensation method, also known as the sol–gel method [37]. Series of samples with the same unit ratios were prepared: T:D = 2:1 and T:D:B = 2:1:1. Triethoxymethylsilane (purity—99%, Merck, Darmstadt, Germany), called unit T, and diethoxydimethylsilane (purity—97%, Merck), called unit D, were chosen as the main substrates in the reaction. However, modified synthesis has been carried out with the use of trimethyl borate (purity—99%, Merck) as the chemical reagent that introduces boron into the system. The preparation of these materials has been executed in controlled conditions under a fume hood; however, this study focused on observing materials’ microstructure and structure changes under the influence of the temperature and atmosphere of the pyrolysis process.

### 2.2. Initial Thermal Treatment and Pyrolysis Process

Products of those syntheses, known as sols, were subjected to an initial thermal treatment at 70–80 °C for a week, resulting in xerogel transformation. Then, xerogels were converted to final materials—SiBOC and SiOC—in the pyrolysis process. This was conducted in three different temperatures—1000, 1200, and 1400 °C—and two atmospheres—argon (Ar) and argon/hydrogen—(ArH—95% Ar, 5%H) for each sample composition.

The following actions were followed to obtain the final materials were followed: The temperatures of the pyrolysis processes were chosen based on thermal studies—TG/DTG—conducted on samples and on our previous experience with such materials [36,38,39]. The pyrolysis process was conducted in a specialized tubular furnace. The temperature increase proceeded with a heating rate of 5 °C/min until 100 °C. After this initial phase, the system was degassed. Following this, the temperature continued to increase with a 5 °C/min rate until reaching the target temperature, which was maintained for 30 min. Finally, the system was cooled down to room temperature. The thermal analysis of the SiBOC and SiOC materials was carried out using Differential Thermal Analysis (DTA) and Thermogravimetry (TG) methods using the STA 449 F3 Jupiter NETSCH analyzer (NETZSCH, Selb, Germany). Measurements were executed on the samples in Al_2_O_3_ crucibles in the argon atmosphere. The temperature range of the analysis was from room temperature to 1300 °C.

### 2.3. Microstructural and Structural Research

#### 2.3.1. Microstructure Analysis—Scanning Electron Microscopy (SEM)

Microstructure research was conducted using a Thermo-fisher Scientific Phenom XL instrument (Waltham, MA, USA) with CeB_6_ as an electron gun. Before measurements, the samples were sputtered with a 7 nm thick layer of gold.

#### 2.3.2. Porosimetry Measurements—Mercury Intrusion Porosimetry (MIP)

Mercury Intrusion Porosimetry research has been carried out using Quantachrome PoreMaster 33 (Boynton Beach, FL, USA). The specimens were subjected to degassing processes at ambient temperature until the pressure reached 10 µm Hg. During measurements, the pressure varied from 0.2 to 33,000 PSI, correlating to pore sizes in the range of 1–7 nm.

#### 2.3.3. Structural Studies—X-Ray Diffraction, FT-IR Spectroscopy, Raman Spectroscopy

X-ray diffraction (XRD) measurements were performed on a PANalytical X’Pert Pro MD diffractometer (Almelo, The Netherlands) equipped with a Ge (111) monochromator with Cu Kalpha1 radiation. The instrument was configured with a ½° divergence slit, a 1° anti-scatter slit on the incident beam, and Soller slits on both the incident and diffracted paths. Data were collected in scanning line detector mode using the X’Celerator detector (PANalytical, Almelo, The Netherlands). During measurement, the sample was rotated with a revolution time of 8 s to ensure improved counting statistics. Phase identification and peak assignment were performed using HighScore Plus 3.0d software (PANalytical, Almelo, The Netherlands) in conjunction with the PDF4+ database.

FT-IR analyses were analyses were carried out using the pellet method using a Bruker Vertex 70v vacuum spectrometer (Billerica, MA, USA). All measurements were conducted in the range of 4000–400 cm^−1^ and with a resolution of 4 cm^−1^, which corresponds to the middle range of infrared radiation. The standard KBr pellet method was employed. Overall, 128 scans were accumulated for each sample.

The WITec Alpha 300M+ spectrometer (Ulm, Germany) was chosen for Raman spectroscopy measurements, which was equipped with a diode laser of wavelength 532 nm. Each sample was studied at 50× magnification via the use of a CCD detector and diffraction grating of 600 slots per millimeter. Each sample was subjected to the measurement of 10 accumulations for 10 s each.

The spectral deconvolution of FT-IR and Raman spectra was carried out using Bruker OPUS 7.2 software. A set of Gaussian–Lorentzian curves with a starting ratio of 50% were set. The root mean square error after the process was found to be below 1 for all deconvolutions.

## 3. Results and Discussion

### 3.1. Thermal Analysis

**Figure 1:**
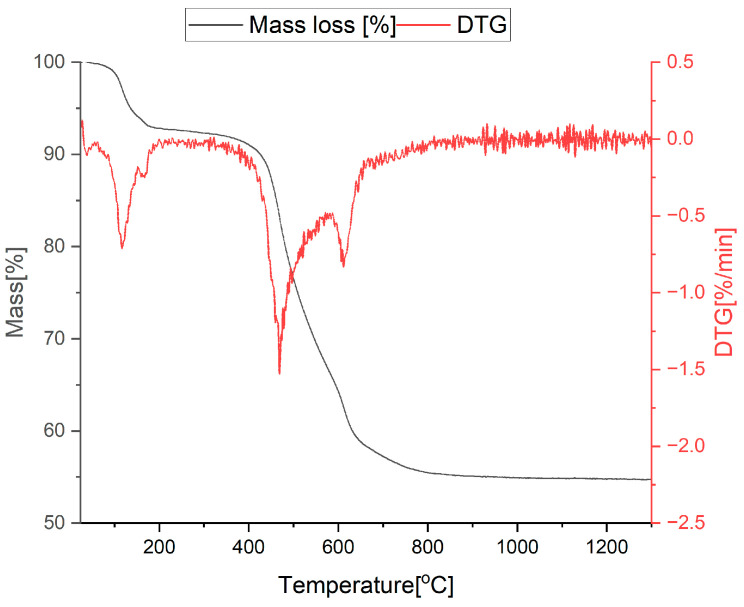
Thermal analysis—TG/DTG curves of sample T:D = 2:1.

**Figure 2:**
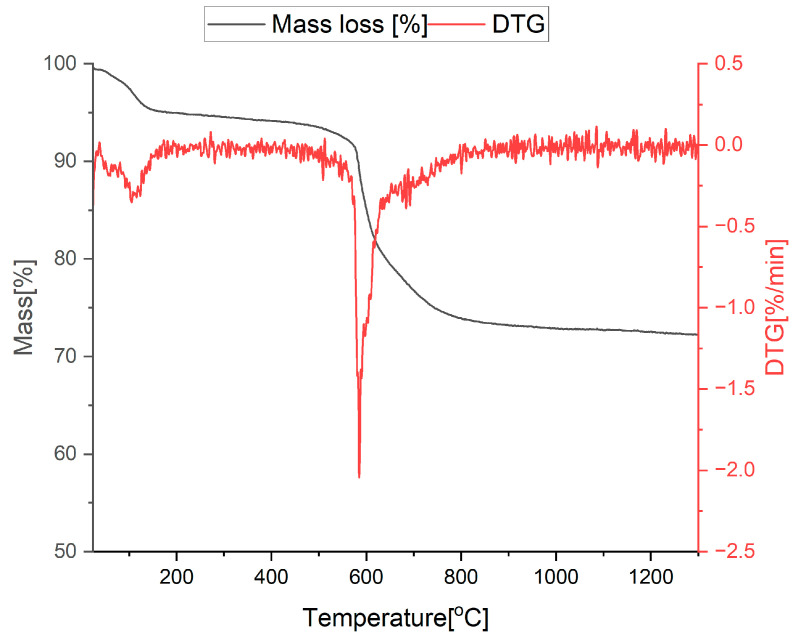
Thermal analysis—TG/DTG curves of sample T:D:B = 2:1:1.

In order to assess the minimum ceramization temperature of the base and modified samples with ratios T:D = 2:1 and T:D:B = 2:1:1, xerogels with the abovementioned proportions were subjected to thermal analysis. As shown in Figure 1, the process of the primary material transformation from xerogel to the final ceramic material happens in stages [38]. However, the highest mass loss occurs between 450 and 650 °C and ends at about 800 °C. Above that temperature, there is no significant change in the sample’s mass. Similar behavior can be observed for boron-doped xerogel, as presented in Figure 2. The stage of higher mass loss happens between 450 and 600 °C and ends at 800 °C. The difference between the base sample and the modified one is related to the amount of mass loss, which, in the base xerogel, equals 45% and, in the modified, equals 25%. The DTG effects on both samples are very similar but are much more prominent for the base sample. Therefore, a 1000–1400 °C range is suitable for the pyrolysis and observation of carbothermal reduction (SiO_2_ (s) + 3C (s) = SiC (s) + 2CO (g)) [40] processes occurring in different atmospheres. Furthermore, it implies that the addition of boron, in the form of trimethyl borate, reduces the weight loss of the material during the pyrolysis process.

### 3.2. Scanning Electron Microscopy

**Figure 3:**
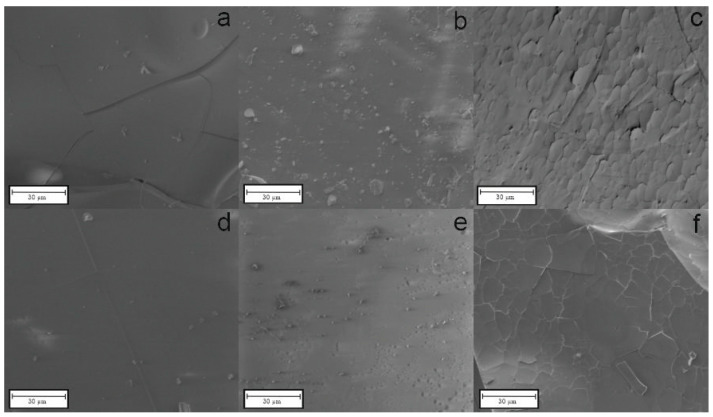
SEM microphotographs of SiOC materials pyrolyzed in (**a**) 1000 °C (**b**) 1200 °C (**c**) 1400 °C in argon/hydrogen atmosphere and (**d**) 1000 °C (**e**) 1200 °C (**f**) 1400 °C in argon atmosphere.

**Figure 4:**
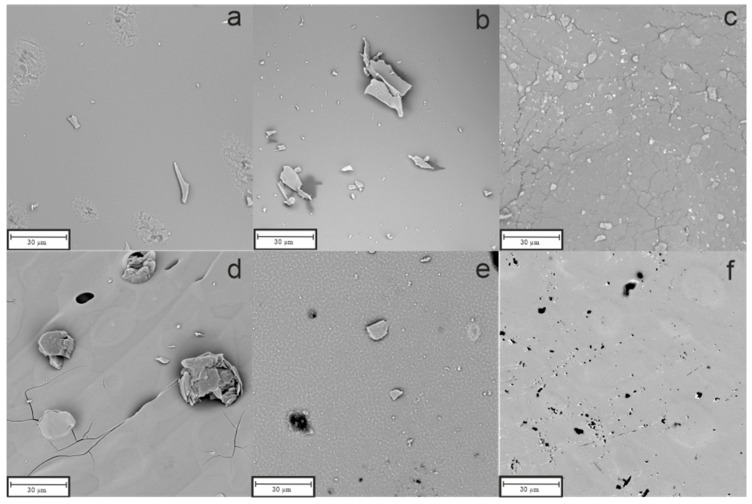
SEM microphotographs of SiBOC materials pyrolyzed at (**a**) 1000 °C, (**b**) 1200 °C, and (**c**) 1400 °C in argon/hydrogen atmosphere and at (**d**) 1000 °C, (**e**) 1200 °C, and (**f**) 1400 °C in argon atmosphere.

**Figure 5:**
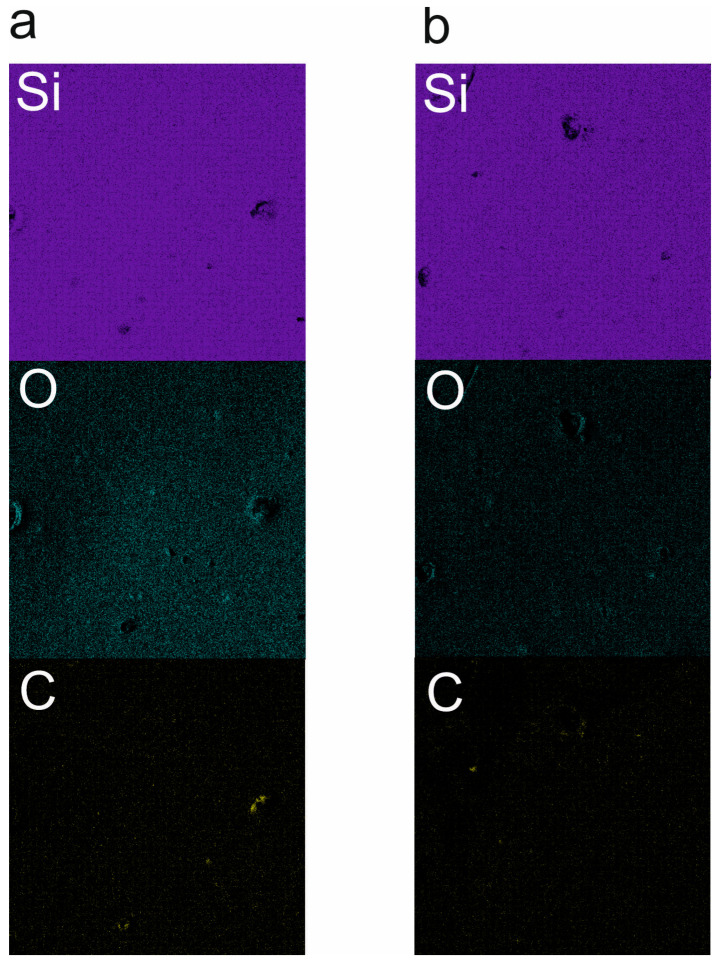
EDS mapping of SiBOC materials pyrolyzed at 1200 °C in (**a**) ArH and (**b**) Ar atmospheres.

Scanning Electron Microscopy (SEM) analysis was carried out to study the microstructure of the pyrolyzed materials. The conducted measurements also involved elemental analysis with Energy Dispersive Spectroscopy (EDS). The results can be found below in Figure 3 (SiOC), Figure 4 (SiBOC), and Figure 5 (SiBOC EDS) for the samples pyrolyzed in different temperatures, in the argon/hydrogen atmosphere, and in the argon atmosphere, respectively.

The base SiOC samples were subjected to microstructure analysis, the results of which are presented in Figure 3. Figure 3a–f show homogenous structures; however, Figure 3c,f (samples prepared in 1400 °C) possess cracks on the surface.

Figure 4a–c show the microstructures of the SiBOC materials obtained in the argon/hydrogen atmosphere at 1000, 1200, and 1400 °C. Pictures taken for the 1000 °C and 1200 °C conditions—Figure 4a,b—present the very similar, almost homogenous, and smooth surfaces of the materials. However, in Figure 4c, where the microstructure of the material obtained at 1400 °C is shown, there are some irregular pores visible at first sight. On the other hand, samples pyrolyzed in the argon atmosphere, as presented in Figure 4d–f, at all temperatures—1000 °C (Figure 4d), 1200 °C (Figure 4e), and 1400 °C (Figure 4f)—are characterized by smooth surfaces with minor imperfections.

Figure 5a,b present the exemplary EDS maps obtained for the SiBOC samples pyrolyzed at 1200 °C in both the ArH (Figure 5a) and Ar (Figure 5b) atmospheres. As can be seen, the distribution of elements is even and very similar for both ceramic specimens (Table 1). Table 1 contains the atomic concentrations of the measured elements for SiOC and SiBOC materials. Due to the nature of EDS measurements, this method is not accurate for elements with low atomic numbers, like C; the percentage given in Table 1 is just a quality number, showing that each of the SiOC elements is present in the given material. Sadly, the same rule applies for boron, as it has a lower atomic number than carbon; hence, it is not detected by our EDS detector. The results of the analysis are supported by the studies presented in a previous article [41].

### 3.3. Mercury Intrusion Porosimetry

**Figure 6:**
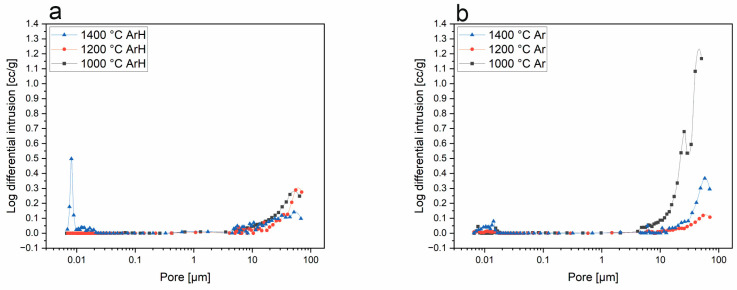
Logarithm differential intrusion—pore size of SiOC samples pyrolyzed in (**a**) ArH and (**b**) Ar atmospheres.

**Figure 7:**
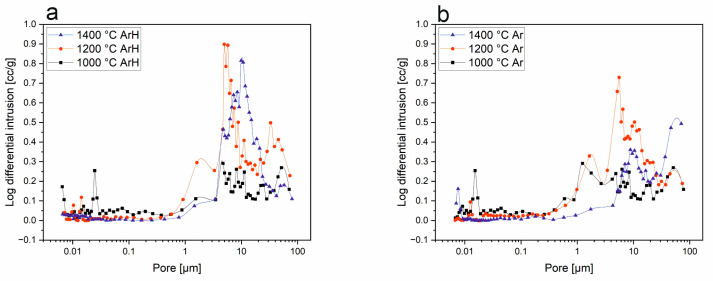
Logarithm differential intrusion—pore size of SiBOC samples pyrolyzed in (**a**) ArH and (**b**) Ar atmospheres.

Figure 6 illustrates the results of the MIP analysis for the base SiOC materials. These samples exhibit very low porosity compared to those that are boron-modified (Figure 7). According to the gathered data, the pores have a diameter above 10 µm.

**Figure 8:**
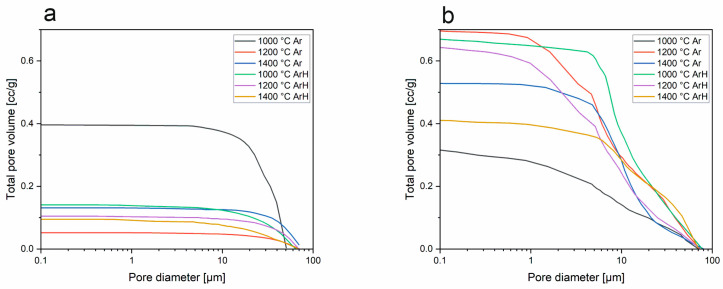
Total pore volume plots of (**a**) base SiOC and (**b**) SiBOC based on MIP measurements.

The porosity, calculated according to the apparent density, was determined to be 53.3%, 56.6%, and 50.1% for the samples pyrolyzed at 1000, 1200, and 1400 °C in the ArH atmosphere and 47.9%, 62.7%, and 59.1% for samples pyrolyzed at 1000, 1200, and 1400 °C in the Ar atmosphere. Based on this analysis, the sample obtained at 1200 °C in argon possesses the highest porosity, equal to 62.7%. In both series of samples, for those pyrolyzed in both atmospheres, the porosity of the samples has a tendency to increase, and the highest porosity was observed at 1200 °C, which then begins to decrease at 1400 °C. Such behavior indicates that in SiBOC samples, the sintering process might take place at temperatures above 1200 °C. While the sintering progresses, the particles bond and densify, which results in porosity reductions. In both the argon/hydrogen and argon samples, pores in the size range of 5–10 µm are dominant, while in those treated at 1400 °C, there are pore sizes above 10 µm, but which are less frequent (Figure 7). This significant difference between SiOC and SiBOC suggests that boron has a substantial influence on the increase in material porosity. As a confirmation of this statement, Figure 8 shows that in SiOC samples, there are barely any pores in the material, while for SiBOC, the highest total pore volume is observed in samples pyrolyzed at 1200 °C in the argon atmosphere.

### 3.4. X-Ray Diffraction

**Figure 9:**
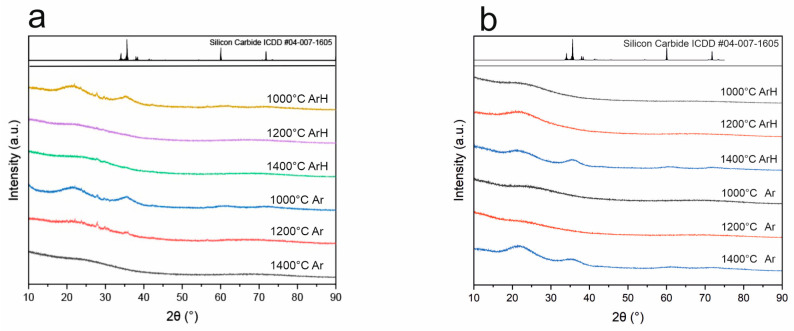
X-ray patterns of (**a**) SiOC and (**b**) SiBOC samples pyrolyzed at different temperatures in ArH and Ar atmospheres.

The structural analysis of ceramic materials is best started with X-ray diffraction. Figure 9 presents the diffraction patterns of the base SiOC and boron-modified samples. All studied materials possess an amorphous structure with characteristic halos shown at 2θ, equal to 22°. However, samples obtained at 1400 °C are characterized by broad peaks of small intensity at 37° that probably correspond to nanocrystalline SiC, with the remaining amorphous halos observed as well at a scattering angle of 22° [20,39,40]. The appearance of such a signal clearly indicates that the carbothermal reduction process occurs in materials only at 1400 °C. This is consistent with the literature where the influence of the temperature has been studied [20]. The conducted studies show that both SiOC and SiBOC materials remain amorphous up to 1200 °C despite different atmospheres.

### 3.5. FT-IR

**Figure 10:**
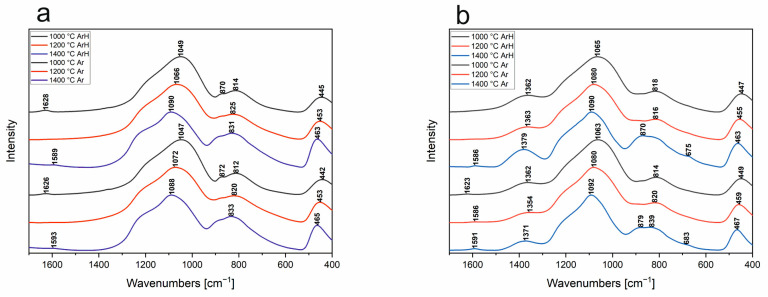
FT−IR spectra in the range 1700–400 cm^−1^ of the (**a**) final SiOC and (**b**) SiBOC materials obtained at different temperatures in the argon/hydrogen and argon atmospheres.

In order to fully describe the structure of the obtained SiOC and SiBOC materials, they are subjected to FT-IR spectroscopic analysis. The spectra shown in Figure 10a,b are typical of PDCs—polymer derived ceramics [41]. The IR spectra presented in Figure 10a,b are similar to each other—the corresponding temperatures in the respective gases. All of the studied materials are characterized by a group of repeating bands—about 1080, 820, and 460 cm^−1^. They originate from stretching vibrations of asymmetric, symmetric, and bending Si-O bonds. This confirms that the structural backbone of the materials is *v*-SiO_2_. In the case of boron-modified materials (Figure 10b), additional bands at about 1360 and 670 cm^−1^ can also be seen to be coming from B-O bond vibrations, confirming the presence of the modifier in the system. For both pure and modified materials, similar trends can be seen on IR spectra—the temperature dependence of the position of bands that are characteristic of silicon–oxygen bonds. Their shift toward higher wavenumbers, as well as their “sharpening”, especially of the bending range, may indicate that the structure ordering is taking place. It is worth mentioning that in Figure 10b, the low intensity band observed at approx. 1590 cm^−1^ indicates the presence of an aromatic C-C bond [42], probably originating from the free carbon phase. This band was observed in the cases of all samples pyrolyzed in the argon atmosphere and the samples obtained at 1400 °C in the argon/hydrogen atmosphere. However, in order to more accurately assess these changes, it is necessary to perform decompositions of the spectra into component bands [43,44,45].

**Figure 11:**
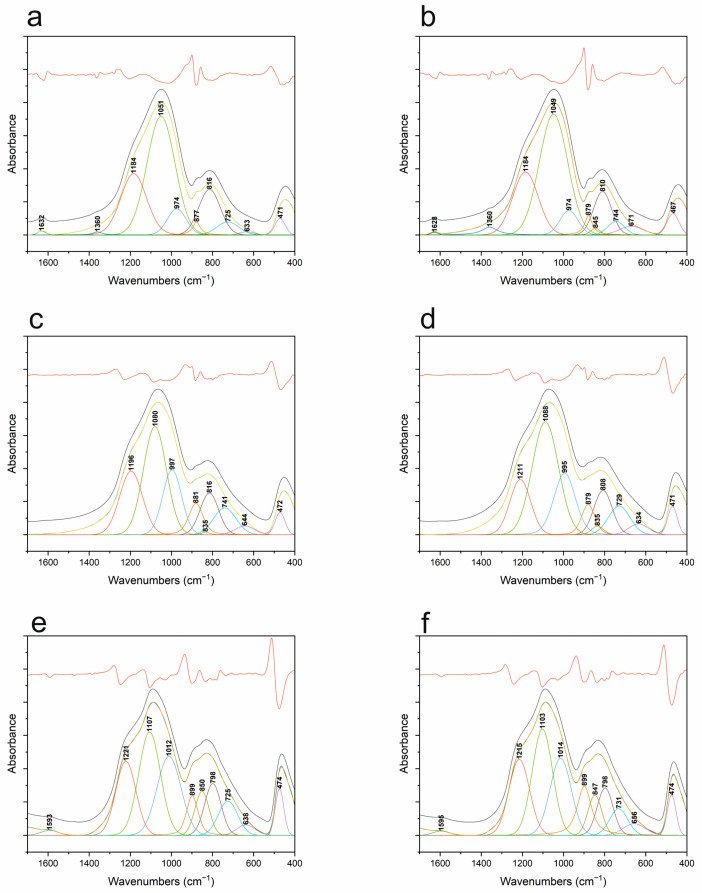
FT-IR spectra decomposition of SiOC samples: (**a**) 1000 °C, ArH; (**b**) 1000 °C, Ar; (**c**) 1200 °C, ArH; (**d**) 1200 °C, Ar; (**e**) 1400 °C, ArH; (**f**) 1400 °C, Ar.

The decompositions carried out (Figure 11) made it possible to determine that each SiOC spectrum consists of the nine main bands described in Table 2. At 1000 °C, organic residues that were not removed at this temperature are still visible. This manifested as the presence of a band at about 1360 cm^−1^, which is responsible for the vibration of Si-CH_x_-Si bridges. In addition, a band at about 1630 cm^−1^ is visible, which is characteristic of H-OH bending vibrations in water adsorbed by the material. For temperatures of 1200 and 1400 °C, organics and adsorbed water are not visible. All of the curve-fitted spectra are characterized by three bands in the range of 900–1300 cm^−1^. They originate from the asymmetric stretching vibration (*v_as_*) of the Si-O bonds (Si-O-, Si-O-Si, and Si=O, respectively). Also typical for the *v*-SiO_2_-based systems is the symmetric stretching vibration observed at about 810 cm^−1^. Similarly, the band at about 471 cm^−1^ is characteristic, originating from O-Si-O bending vibrations. The positions and half-widths of these mentioned bands strongly depend on temperature. As it increases, a shift toward higher wave numbers of the asymmetric stretching bands can be seen. This indicates that the material structurally approaches amorphous silica, whose typical band originating from *v_as_* Si-O-Si occurs at about 1100 cm^−1^. However, despite the occurrence of this convergence, there are no signs of SiO_2_ crystallization in the studied materials, as confirmed by XRD. What can be seen, however, is a change in the intensity of the band at around 845 cm^−1^, characteristic of SiC. At 1400 °C, this band becomes definitely visible—an increase in its intensity. This may confirm the nanocrystallization seen in XRD diffractograms.

**Figure 12:**
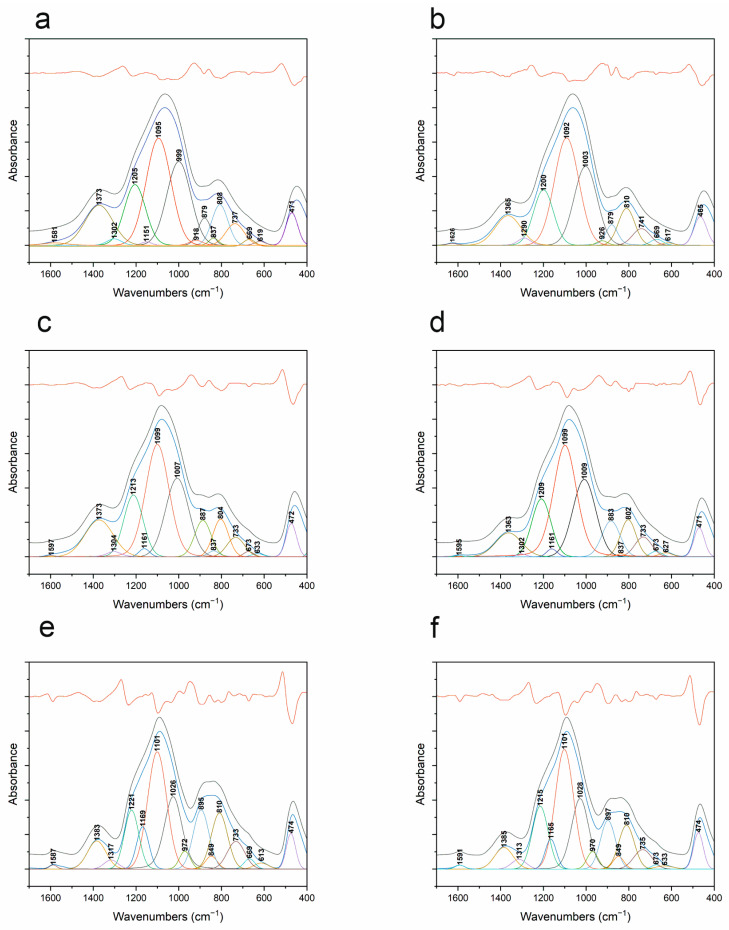
FT-IR spectra decomposition of SiBOC samples: (**a**) 1000 °C, ArH; (**b**) 1000 °C, Ar; (**c**) 1200 °C, ArH; (**d**) 1200 °C, Ar; (**e**) 1400 °C, ArH; (**f**) 1400 °C, Ar.

Due to the complex nature of the obtained FT-IR spectra (bands with large half-width), the precise determination of the structure of the studied materials is practically impossible. Therefore, it was necessary to decompose the raw spectra into component bands—Figure 12 and Table 3. The analysis made it possible to determine that, with increasing temperature, the band responsible for asymmetric stretching vibrations of Si-O-Si shifts from its position at about 1090 (1000 °C) to about 1100 cm^−1^ (1400 °C) for each atmosphere. This shift is smaller than that observed without decomposition, where other elements affecting the shape of the spectral envelope also have an influence. This shift in band position is probably due to the formation of domains typical of *v*-SiO_2_ [36,46], since, at 1400 °C, the carbothermal reduction in SiOC to SiO_2_ and SiC should already be occurring. This is also indirectly indicated by the XRD analysis, where nanocrystalline SiC is probably visible, but where there are no other signs of the process in question. At the same time, IR spectra do not show the typical low half-width bands of crystalline compounds. Therefore, it can be assumed that the process, if it occurs, occurs on a very limited scale. The decompositions of the spectra into component bands also make it possible to accurately attribute the presence of boron with coordination numbers (CN) 3 and 4. The presence of boron in CN 3 is confirmed by bands at about 1380, 730, and 670 cm^−1^ [22]. The former is responsible for B-O stretching vibrations in BO_3_ units, while the latter is responsible for B-O-B vibrations between BO_3_ and BO_4_. The last band can be assumed to be responsible for B-O-B vibrations in BO_3_ units. These bands are present for all temperatures regardless of the atmosphere used. This confirms the presence of boron in CN 3. At the same time, bands characteristic of boron in CN 4 are visible at 1290, 1150, and 880 cm^−1^. The intensity of these bands, especially 880 cm^−1^, increases with the increasing temperature. This indicates that boron coordination changes as a function of temperature. This is also confirmed by the presence of bands typical of Si-O-B junctions, as in classical borosilicate glasses, at about 1000 cm^−1^ and 630 cm^−1^ [43]. On the one hand, the results indicate that the boron present in CN 3 may be a separate matrix of the glass, which merges with the SiOC matrix after increasing the temperature. This results in a transition to an oxyborosilicate matrix, in which boron is also present in CN 4. Confirmation of the SiOC structure itself can be found at the 840 cm^−1^ wavenumber—the presence of a band characteristic of Si-C vibrations in silicon oxycarbide [45]. XRD analyses suggested the presence of nanocrystalline silicon carbide. However, the conducted spectral deconvolution does not confirm its presence. This means that, from the spectroscopic point of view, all of the tested samples are amorphous.

Comparing the decomposed IR spectra of the base materials together with the modified ones, it can be seen that the introduction of boron causes the band characteristic of the asymmetric stretching vibrations of Si-O-Si already at 1000 °C to be shifted to about 1090 cm^−1^. Its shift as a function of temperature is much smaller than for the undoped material. At the same time, the effect is the same—the band shifts to the position typical of *v*-SiO_2_. In both this and the other case, XRD indicates an ongoing process of crystallization, or rather nanocrystallization, of SiC, which can probably be partially confirmed by FT-IR spectroscopy for the SiOC material.

### 3.6. Raman Spectroscopy

As a complementary method to FT-IR spectroscopy, Raman measurements have been carried out on the SiOC and SiBOC materials. Raman spectroscopy, due to its nature, is very sensitive to the presence of a carbon phase. Typically, there are two main, also called first-order, scattering bands related to the carbon structure, which appear for almost all studied samples in both argon/hydrogen and argon atmospheres. For the SiOC and SiBOC samples pyrolyzed at 1000 °C in Ar and ArH, only fluorescence is visible, except for the ArH SiBOC sample. This is a common feature for oxycarbide materials, especially for the ladder-like derived silsesquioxane ones [47]. Only after reaching a certain temperature, the free carbon phase becomes visible.

**Figure 13:**
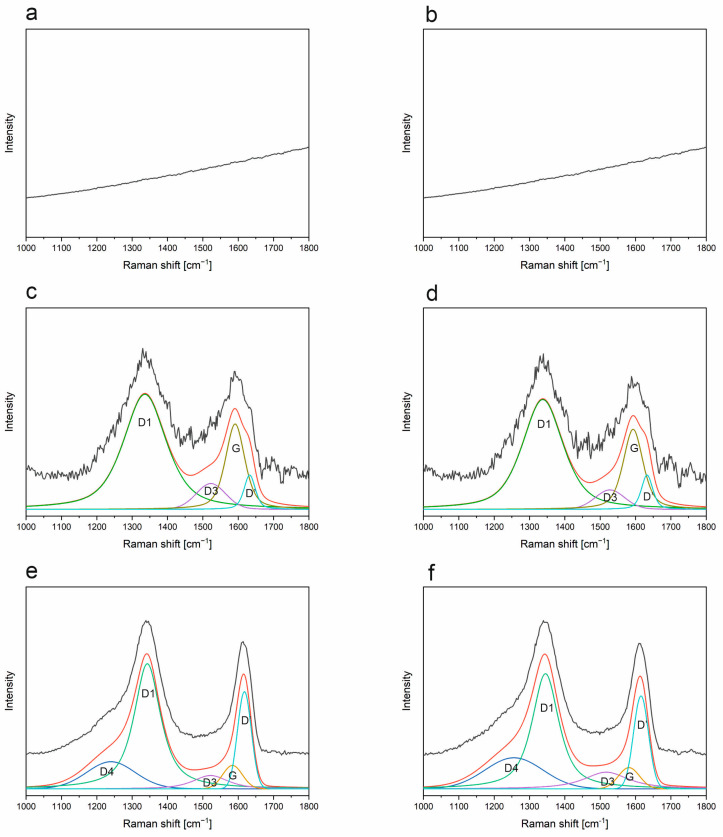
Deconvoluted Raman spectra of SiOC samples pyrolyzed in selected temperatures (**a**) 1000 °C, ArH; (**b**) 1000 °C, Ar; (**c**) 1200 °C, ArH; (**d**) 1200 °C, Ar; (**e**) 1400 °C, ArH; (**f**) 1400 °C, Ar.

**Figure 14:**
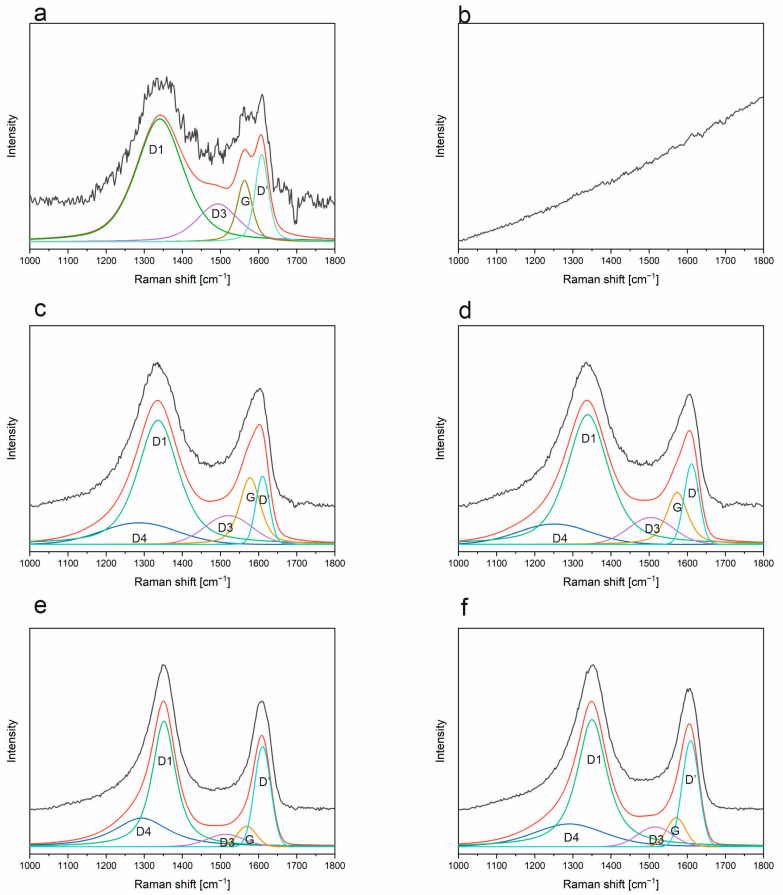
Deconvoluted Raman spectra of SiBOC samples pyrolyzed in selected temperatures: (**a**) 1000 °C, ArH; (**b**) 1000 °C, Ar; (**c**) 1200 °C, ArH; (**d**) 1200 °C, Ar; (**e**) 1400 °C, ArH; (**f**) 1400 °C, Ar.

All recorded spectra (Figure 13 and Figure 14) have been cut to show the 1000–1800 cm^−1^ range, as only bands related to first-order scattering are visible due to ongoing fluorescence. The bands at approx. 1360 cm^−1^ and 1600 cm^−1^ correspond to D-band and G-band, respectively [30,48]. The first one can be described as the “defect-induced band”, associated with the breathing vibrations of hexagonal carbon rings, while the latter can be related to ordered graphitic lattices, assigned to the stretching of the hexagonal carbon rings [49].

Base SiOC samples, as well as SiBOC ones, have been subjected to the deconvolution process, as described in Materials and Methods, and the analysis is presented in Figure 13 and Table 4 for the base materials and Figure 14 and Table 4 for the modified ones. The mathematical analyses carried out made it possible to assign the following band positions: D1, D3, D4, D’, and G bands at approx. 1350, 1540, 1250, 1610, and 1570 cm^−1^, respectively [50,51,52]. The reported positions change with the temperature of pyrolysis as well as the incorporation of boron modification. The D1 band is the abovementioned D band defect-activated breathing mode of sp^2^ rings. The D3 can most probably be assigned to amorphous carbon, while D4 is a remnant of sp^3^ C-C vibrations or some disordered carbon structures. In the case of the D4 band, the first option seems most likely to be the preceramic materials used, which are ladder-like silsesquioxanes with methyl functional groups. The G band—the in-plane stretching of sp^2^ carbon is connected with the D’ band—encompasses the defect-related graphene edge vibrations. The analysis of the base material at a temperature of 1000 °C indicates that no carbon bands are present in either atmosphere. The fluorescence present in the system makes it impossible to record the correct Raman spectrum. This is probably due to the presence of organic residues that did not manage to transform into the carbon phase at this temperature, as confirmed by the FT-IR results and the presence of a band at about 1350 cm^−1^ (Figure 10). When the temperature is increased to 1200 °C, typical carbon bands appear, but their intensity and signal-to-noise ratio are very weak. In addition, fluorescence is still visible and strongly affects Raman measurements (Figure 13c,d). The spectra of the samples obtained at 1200 °C are characterized by the same number of bands regardless of the gas used. The spectra are almost identical, indicating that regardless of the gas, the carbon phase obtained is the same. Typical D1 and G bands, which have the highest intensity, are present. Also visible are a D’ band from edge defects and a small D3 band, indicative of an amorphous carbon phase. For samples obtained at 1400 °C, an additional band—D4—becomes visible. In addition, the half-width of the D1 band changes, and, instead of the G band, the D’ band begins to dominate. This is a change that is quite surprising. It indicates the generation of a significant number of edge defects as the temperature increases. And, on the other hand, the change in the half-widths of the D1 and D’ bands may indicate the appearance of carbon phase ordering, but only in the nanometer range.

In the case of SiBOC materials derived from polymeric ladder-like structures, the situation is different. As can be seen in Figure 14a, as well as in Table 4, these bands have similar intensities (I), as well as Integrals (II), while still keeping the proportions reversed. As the temperature rises to 1200 °C (Figure 14c,d), the G band is still similar in I and II to the D’ one. At 1400 °C, the G band experiences a drop in both I and II compared to previous temperatures. The prominence of the D’ band indicates a significant contribution from defect-induced scattering or graphene edge vibrations. This is often observed in highly disordered carbons or materials with a high density of defects like nanocrystalline graphite. A weaker G band is a typical feature in less ordered carbons or when sp^2^ domains are very small. The behavior of the D3 band is similar to that of G, which is obvious since the higher temperature should eliminate the amorphous phase. Also, a change in the full width at half maximum (FWHM) for the D1 and D’ bands is observed with the changing temperature (Table 4). This, combined with the above discussed changes in the G band, may suggest that edge defects are becoming more localized [53,54,55,56,57,58]. As described, the shift in intensities of G and D’ reflects a higher defect density and reduced crystallinity and/or smaller sp^2^ domains in the carbon material. This is consistent with materials that have undergone significant structural modification or mechanical processing [53]. In this current case, calculating the I_D_/I_G_ ratio is pointless because the values obtained are not from the scale used to characterize carbon materials (Ferrari–Robertson Diagram).

Comparing the base materials and those modified with boron, it can be seen that in the latter, the D’ band appears as early as 1000 °C and is the dominant band over the G band all the time, regardless of the gas used. This shows that the addition of boron affects the generation of edge defects in the free carbon phase. Also, the same addition has an effect on reducing the fluorescence of the whole system and on improving the signal-to-noise ratio, which manifests itself in a better quality spectrum at temperatures of 1000 °C. The prominent share of the D4 band in the spectra of the base materials obtained at 1400 °C may indicate that a confirmation of the presence of disordered carbon structures, since it always occurs in tandem with the D’ band.

## 4. Conclusions

The impact of the temperature and different ceramization atmosphere on the structure and microstructure of SiOC-based materials was evaluated. Two series of samples have been prepared and closely analyzed—base SiOC and boron-modified SiBOC materials. The materials were obtained under the same conditions, starting from synthesis to the initial and final thermal treatment.

The conducted studies revealed the following:

(a) SEM with EDS studies revealed homogenous material surfaces in all samples.
(b) Porosity studies indicated that SiBOC materials regarding temperature and atmosphere were porous, with the highest porosity in samples treated at 1200 °C under Ar. However, SiOC samples do not possess pores in the structure. MIP studies suggest that TMB, which was the reagent introducing boron to the system, can be a porosity initiator.
(c) XRD studies revealed that both SiOC and SiBOC materials remain fully amorphous up to 1200 °C, and at 1400 °C, samples pyrolyzed in both ArH and Ar exhibited amorphous structures, probably with a nanocrystalline SiC phase. These results suggest that the crystalline structure of the material depends on the pyrolysis temperature and that the atmosphere does not have an impact as long as it is inert.
(d) FT-IR detailed studies showed the temperature dependence of chemical structure changes in SiBOC materials. With its increase, boron moved to a coordination number of 4, forming an oxyborosilicate matrix. FT-IR measurements of SiOC materials partially confirmed the nanocrystalline phase of Si-C in samples pyrolyzed in 1400 °C.
(e) The presence of the free carbon phase was detected using Raman spectroscopy for samples obtained above 1000 °C. The free carbon phase was identified in different forms—sp^2^ and sp^3^. The amount of defects and fluorescence depends on the addition of boron.
(f) Both ArH and Ar atmospheres do not affect the chemical structure of the SiBOC matrix, but their effect on the microstructure is significant. On the other hand, the effect on base SiOC materials is negligible.
(g) The free carbon phase is dependent on both the temperature of pyrolysis and the atmosphere. With increasing temperature, the intensity of the G band decreases (ordered sp^2^ domains decrease), the D’ band intensity increases (defects increase), and the FWHM of both D1 and D’ decreases (defects become more localized).

It can be stated that the addition of boron in SiOC materials can serve to control the porosity of the system. At the same time, the introduction of boron causes greater defect generation in the free carbon phase and, depending on the gas used, also changes the microstructure of the material. The addition of boron influences the conversion of the silicon–oxygen matrix of pure SiOC into a mixed silicon/boron–oxygen and borosilicate matrix of SiBOC.

## Figures and Tables

**Table 1:** Atomic concentration of SiOC pyrolyzed samples.

Atomic Concentration [%]			
	Si	O	C
1000 °C ArH SiOC	43.26	50.50	6.25
1000 °C Ar SiOC	45.17	51.82	3.01
1200 °C ArH SiOC	47.57	49.30	3.13
1200 °C Ar SiOC	43.68	53.61	2.71
1400 °C ArH SiOC	40.85	51.50	7.64
1400 °C Ar SiOC	41.23	53.56	5.22
1000 °C ArH SiBOC	39.72	42.53	17.75
1000 °C Ar SiBOC	31.62	59.34	9.05
1200 °C ArH SiBOC	29.46	39.90	30.64
1200 °C Ar SiBOC	32.34	45.84	21.83
1400 °C ArH SiBOC	33.06	43.57	23.37
1400 °C ArH SiBOC	29.79	57.19	13.02

**Table 2:** FT-IR band assignment of deconvoluted spectra [43,44,45].

Band Position [cm^−1^]						Vibration Origin
1000 °C Ar	1000 °C ArH	1200 °C Ar	1200 °C ArH	1400 °C Ar	1400 °C ArH	
467	471	471	472	474	474	δ O-Si-O
671	633	634	644	656	638	S4R + S6R 4 and 6 membered rings
744	725	729	741	731	725	S3R 3 membered rings
810	816	808	816	798	798	*v_s_* Si-O-Si
845	-	835	835	847	850	*v* Si-C
879	877	879	881	899	899	δ Si-H
974	974	995	997	1014	1012	Si-O^−^
1049	1051	1088	180	1103	1107	*v_as_* Si-O-Si (C)
1184	1184	1211	1196	1215	1221	*v_as_* Si-O-Si (Si=O)
1360	1360	-	-	-	-	*v* Si-CHx-Si
1628	1632	-	-	-	-	δ H-O-H
-	-	-	-	1595	1593	*v* C-C

**Table 3:** FT-IR band assignment of deconvoluted spectra of SiBOC materials [43,44,45].

		Band Position [cm^−1^]				Vibration Origin
1000 °C Ar	1000 °C ArH	1200 °C Ar	1200 °C ArH	1400 °C Ar	1400 °C ArH	
465	468	469	469	474	474	δ O-Si-O
616	617	628	632	632	614	δ B-O-Si
670	668	672	669	673	669	δ B-O in BO_3_
741	735	732	733	735	733	δ BO_3_-O-BO_4_
811	805	803	804	810	810	*v_s_* Si-O-Si
838	834	837	837	849	849	*v* Si-C
880	877	884	887	897	895	δ Si-H/*v* BO_4_
925	916	-	-	-	-	δ Si-OH
-	-	-	-	970	972	B-O-B or B-O-Si
1002	998	1009	1007	1028	1026	Si-O^−^ or B-O-Si/pentaborate/Si-O^−^
1091	1093	1099	1100	1101	1101	*v_as_* Si-O-Si
1148	1149	1160	1161	1165	1169	*v* B-O in BO_4_
1200	1203	1209	1212	1215	1221	Si-O-Si
1289	1300	1303	1303	1313	1317	BO_4_
1365	1371	1363	1373	1385	1383	*v* B-O in BO_3_
-	-	1595	-	1591	1587	*v* C-C
1626	-	-	-	-	-	δ H-O-H in H_2_O

**Table 4:** Raman deconvolution—report data.

Sample	Band	Raman Shift [cm^−1^]	Intensity [a.u.]	FWHM [cm^−1^]	Integral Intensity [a.u.]
1000 °C ArH SiBOC	D1	1341	1.69	146	320.62
	D4	1493	0.52	128	87.40
	G	1563	0.84	47	51.87
	D′	1608	1.20	41	63.99
1200 °C ArH SiBOC	D4	1287	0.29	236	73.69
	D1	1336	1.70	127	293.95
	D3	1521	0.39	145	60.63
	G	1577	0.91	65	77.98
	D′	1610	0.93	42	42.49
1200 °C ArH SiOC	D1	1335	1.7	137	309
	G	1554	0.6	181	134
	D′	1603	1.0	74	90
1200 °C Ar SiBOC	D4	1251	0.28	226	66.49
	D1	1339	1.78	128	310.64
	D3	1504	0.37	139	53.97
	G	1573	0.71	65	64.05
	D′	1611	1.10	46	54.57
1200 °C Ar SiOC	D1	1335	1.6	123	262
	G	1554	0.6	181	134
	D′	1605	0.9	67	83
1400 °C ArH SiBOC	D4	1293	0.39	170	98.14
	D1	1352	1.73	73	183.95
	D3	1513	0.17	115	22.63
	G	1569	0.28	59	17.69
	D′	1610	1.38	51	74.12
1400 °C ArH SiOC	D4	1235	0.4	156	59
	D1	1343	1.9	91	268
	G	1562	0.3	108	42
	D′	1616	1.5	48	76
1400 °C Ar SiBOC	D4	1291	0.31	221	85.23
	D1	1350	1.75	93	240.15
	D3	1516	0.27	109	31.37
	G	1571	0.40	58	24.67
	D′	1609	1.46	52	80.25
1400 °C Ar SiOC	D4	1242	0.4	175	68
	D1	1345	1.9	95	273
	G	1553	0.3	118	50
	D′	1613	1.4	52	80

## Data Availability

The original contributions presented in this study are included in the article. Further inquiries can be directed to the corresponding author.
